# Women with Type 1 Diabetes Mellitus: Effect of Disease and Psychosocial-Related Correlates on Health-Related Quality of Life

**DOI:** 10.1155/2018/4079087

**Published:** 2018-05-03

**Authors:** Syed Wasif Gillani, Irfan Altaf Ansari, Hisham A. Zaghloul, Mohi Iqbal Mohammad Abdul, Syed Azhar Syed Sulaiman, Mirza R. Baig, Hassaan Anwar Rathore

**Affiliations:** ^1^College of Pharmacy, Taibah University, Al Madinah Al Munawarah, Saudi Arabia; ^2^Department of Pathology, College of Medicine, Taibah University, Al Madinah Al Munawarah, Saudi Arabia; ^3^School of Pharmaceutical Sciences, Universiti Sains Malaysia (USM), Penang, Malaysia; ^4^Dubai Pharmacy College, Dubai, UAE

## Abstract

**Background:**

This study is aimed at investigating the various disease-specific and health-related psychosocial concepts of HRQOL among insulin-dependent diabetes mellitus (IDDM) and understanding the gender differences in HRQOL among IDDM patients.

**Methods:**

A cross-sectional observational study was conducted to assess the effect of health-related and psychosocial correlates on HRQOL of IDDM patients in Penang, Malaysia. The participants were recruited from five governmental diabetic clinics. Patients with insulin use only, IDDM diagnosed at least 1 year earlier, were identified from clinical registers. The sample was then age stratified for 20–64 years, and severe complications (e.g., end-stage renal failure, hemodialysis, and liver cirrhosis) were excluded; a total of 1003 participants were enrolled in the study. Multivariate regression analysis was used to predict the response.

**Results:**

A total of 853 (100%) participants were enrolled and completed the study. Women exhibited significantly higher/better mental health (*p* < 0.013) and health perception scores (*p* < 0.001) despite high prevalence of impaired role (49.2%), social (24.2%), and physical (40.5%) functionings as compared to men. Women with longer diabetes exposure and uncontrolled glycemic levels (HbA1c) have poorer HRQOL. Availability of social support showed no significant association with either HRQOL or diabetes distress levels. Diabetes distress levels remained not associated with social support. Women also showed significantly higher association with health perception (15% versus 13% men, *p* < 0.001) and mental health (13% versus 11% men, *p* < 0.001) in diabetes-specific psychosocial factors. Thus, among women alone, diabetes-related specific and psychosocial factors explained 15% and 13% of variations in HRQOL extents, respectively.

**Conclusion:**

Women exhibit extensive and significant patterns with health-related factors and diabetes-specific psychosocial factors (self-efficacy, social support, and DLC) to improve HRQOL. Also, women have significantly high reported distress levels and low social functioning compared to men.

## 1. Introduction

Diabetes mellitus (DM) is a metabolic disorder categorized with relative (type 2) and absolute (type 1) deficiency of glucose regulatory hormone—insulin [1]. More than three hundred fifty million people were recognized with DM by 2011 and estimated to be doubled by 2030 [2]. Insulin-dependent diabetes mellitus (IDDM/type 1 DM) is presented with either lacking or reducing of insulin segregation or completely depletion of pancreatic cells responsible for producing insulin. Thus, the relevant patients required daily parenteral dosing of insulin to maintain glycemic levels. Disease progression rate is directly related to glycemic control; poor glycemic control leads to rapid disease progression and therefore involves in serious complications: nephropathy, retinopathy, macrovascular diseases, etc. [3, 4]. Thus, treatment of IDDM is based on monitoring and delaying harmful effects by controlling glycemic levels and improving relative quality of life (QOL) of the patient.

Self-care is the one of the important factor for achieving optimal glycemic levels among patients with IDDM [2, 3]. Diabetes patients are responsible for their daily care, such as self-monitoring blood glucose (SMBG), diet management, and insulin dose adjustments [5, 6]. Diabetes affects the daily life functioning therefore, lowering the health status and QOL among patients [1, 2]. Such factors cause treatment failure and poor glucose tolerance which in turn leads to poorer health quality of life (HRQOL) [7].

Treatment success or failure also based on patient's satisfaction to different aspects of disease domains [1, 6]. Diabetes is often examined in terms of compliance and metabolic outcomes. Clinical comorbidities play an important role in diabetes progression and also provide sufficient information for patient-treatment adjustments [8]. Therefore, in recent years, the scientific literature focused on evaluating HRQOL among patients with chronic disorders.

HRQOL is a multidimensional construct [9] comprising of physical, social, and psychosocial domains. Also, the core feature of HRQOL is the self-evaluation of patient regarding his health-related quality of life [1, 4, 6]. Studies have suggested that adequate self-care will improve the glycemic control [1, 3]. Certainly, internal locus of control of having a belief that life events are resultant of past own actions would help and beneficial for patient's active disease coping [1, 7]. Social/peer support also considered as an important factor in controlling disease-related psychosocial problems and improving health literacy together with modified psychosocial factors such as beliefs about disease and treatment [4, 10]. Thus, to prevent future diabetic complications, it is necessary to understand the factors relating to the HRQOL among patients with chronic illnesses to reduce the risk of severe functional limitations.

The aim of this study was to investigate the various disease-specific and health-related psychosocial concepts of HRQOL among IDDM patients, especially on social support, diabetes-related psychosocial factors, and self-care behavior and practices to understand the gender differences in HRQOL among IDDM patients.

## 2. Methodology

### 2.1. Ethical Approval

Approvals were made prior to conducting the study from Ministry of Health, Malaysia and Clinical Research Committee (CRC), registration ID: NMRR-17-776-6941.

### 2.2. Study Design, Location, and Duration

A cross-sectional observational study was conducted to assess the effect of health-related and psychosocial correlates on HRQOL of IDDM patients in Penang, Malaysia. The participants were recruited from five governmental diabetic clinics. Participant recruitment process was done from February to March 2017, data collection from April to June 2017, and analysis from July to September 2017. Written consent forms were obtained from all the participants.

### 2.3. Recruitment Process

**Figure 1:**
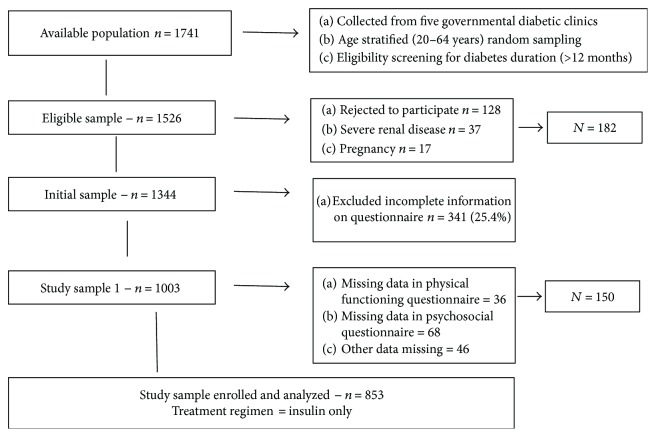
Recruitment process and the study.

Patients with insulin use only, IDDM diagnosed at least 1 year earlier, were identified from clinical registers. The sample was then age stratified for 20–64 years, and severe complications (e.g., end-stage renal failure, hemodialysis, and liver cirrhosis) were excluded; a total of 1526 participants were eligible to participate. Out of them, 1344 patients were approached/agreed to participate by post, mail, telephone, or during follow-up visits. The recruitment process is briefly explained in Figure 1. A total of 853 participants' data were used to analyze the hypothesis of the study from the eligible sample (1344). Participants with missing data and nonrespondents were analyzed to evaluate the differences between age, gender, comorbidities, and glycemic control values (recent) from the clinical records. No significant difference was found in these variables (data is not presented).

This study presented the subsample of participants with age 20–64 years old, insulin treatment only, without severe complication (no effect of HRQOL), attended the clinic for clinical assessments (HbA1c), and completed/returned all the questionnaires for the study.

## 3. Instruments

### 3.1. Dependent Measures

Health-related quality of life (HRQOL) was measured by the prevalidated Malaysian version of the medical outcome study (MOS-SF-20 [11, 12]) health survey questionnaire. There are six self-reported subscales; higher value indicates better well-being. A pilot study (*n* = 68) among Malaysian IDDM s was conducted to validate subscales as health perception (5 items, Cronbach's *α* coefficient = 0.89), physical functioning (6 items, *α* = 0.85), role functioning (2 items, *α* = 0.94), mental health (5 items, *α* = 0.91), social functioning (1 item), and pain (1 item). Reported physical functioning was used as a control variable in this study.

### 3.2. Independent Measures

*Demographics*: the variables were age, marital status, education, and occupation status.

*Health-related factors*: diabetes exposure, comorbidities, glycemic control (HbA1c), and physical functioning reported at MOS-SF-20.

*Social support*: the measure for general support was adopted from the literature [13]. Participants have to assess the support with adequacy and/or availability. Internal consistencies for the composite scales were 0.71 and 0.88 with adequacy and availability.

*Diabetes-related specific support* [14, 15]: the original 16-item scale was modified for this study on the basis of measures with supportive family behavior only (excluding peer/friends). The alpha coefficient values were constructed by using factor analysis (component analysis-varimax rotation). Supportive behaviors: (9-items, *α* = 0.79 and criticizing behavior: 7 items, *α* = 0.75. The diabetes-related specific support measure was then modified by subtracting the criticizing behavior from composites.

*Diabetes-related health beliefs* [16, 17]: perceived benefits of the regimen (8 items, *α* = 0.89).

*Susceptibility scale* [18, 19]: participants estimated the likelihood of a diabetes-related health hazards (e.g., eye disease, pain in feet, kidney disease, and ambulation). Each measures on 5-point Likert scale (*α* = 0.91). Severity of these complications was estimated on 7-point Likert scale (*α* = 0.88).

*Diabetes locus of control (DLC)* [20]: a 27-item scale with four subscales was used; internal DLC (*α* = 0.79), chance DLC (*α* = 0.76), professional DLC (*α* = 0.80), and other DLCs (*α* = 0.71).

*Self-efficacy scale* [21]: a 13-item measure for perceived competence in self-care with overall alpha coefficient (*α* = 0.86) was used to evaluated self-report on dietary habits, frequency of exercise, and self-monitoring blood glucose (SMBG).

*Problem areas in diabetes (PAID)* [22]: a 20-item measure (*α* = 0.93) was used to estimate the participants' response on a 5-point Likert scale. Total score ranges between 0 and 100; higher value indicates high distress.

*N.B.*: construction and psychometric validation of health beliefs, susceptibility, DLC, self-efficacy, and PAID scales were evaluated in the pilot study as mentioned earlier.

### 3.3. Statistical Analysis

Bivariate and multivariate models were used to determine the association between gender and demographic factors and independent variables. Hierarchical regression analysis was used to determine the predictive association of diabetes-related psychosocial factors on HRQOL. Health beliefs, mental health status, and diabetes distress associated with HRQOL were evaluated with logistic regression modelling. Also, role and social functioning and pain measures were dichotomized for logistic regression analysis because of the skewed distribution pattern.

The stepwise multiple regression analysis was used in the following order: step 1: sociodemographic, step 2: health-related factors, step 3: social support, step 4: diabetes-related support, step 5: diabetes-related health beliefs, and step 6: self-care practices. The final regression model was presented after adjustment of background factors. Initial logistic model starts with steps 1–4 and then followed by subsequent steps.

## 4. Results and Findings

### 4.1. Sample/Participants' Characteristics

A total of 853 (100%) participants were enrolled and completed the study; Table 1 presented the sample characteristics. Men to women ratio was 1.05 : 1. Women showed significantly higher mean age (*p* < 0.001) with low duration of diabetes exposure (years) (*p* < 0.021) than men. Findings also suggested that women exhibited significantly higher/better mental health (*p* < 0.013) and health perception scores (*p* < 0.001) despite high prevalence of impaired role (49.2%), social (24.2%), and physical (40.5%) functionings as compared to men. In contrast, women (64.9%) reported pain was significantly (*p* < 0.001) higher than men (43.1%). Also, the diabetes distress scale score showed that women have significantly higher (*p* < 0.011) mean score (48.1) than men (36.4) which reflects emotional burnout.

### 4.2. Sociodemographic Characteristics and HRQOL: Association/Pattern

In the analysis, both genders reported a similar pattern with age, as increase in age significantly related with reduced/poorer HRQOL in health perception (*p* < 0.05) and pain (*p* < 0.001). Women showed more difficulty in role (*p* < 0.01) and social (*p* < 0.05) functionings as compared to men. Women who were not currently in married status have more difficulty in physical functioning (*p* < 0.001) and also lower mental health scores (*p* < 0.05). Findings also suggested that the lower education status of women relates to high distress levels (*p* < 0.001), pain (*p* < 0.001), and role functioning (*p* < 0.01). The longer diabetes exposure and uncontrolled glycemic levels (HbA1c) of women are related to poorer HRQOL with health perception (*p* < 0.01), mental health (*p* < 0.001), physical (*p* < 0.01), and social (*p* < 0.001) functionings and increase distress levels (*p* < 0.001). Bivariate analysis is provided in Table 2.

### 4.3. Social Support and HRQOL

Availability of social support showed no significant association with either HRQOL or diabetes distress levels. However, perceived adequacy of social support showed significant association with HRQOL in all subvariables except role and social functionings. Diabetes distress levels remained not associated with social support.

### 4.4. Disease-Related Psychosocial Factors and HRQOL

Younger age women with married status correlates increase self-efficacy (*p* < 0.001) in diabetes management and strong diabetes social support (*p* < 0.001) with reduce diabetes distress score (*p* < 0.001) than men; in contrast, older age (regardless of marital status) women's longer diabetes exposure and increase comorbidities are related with poorer HRQOL in health perception (*p* < 0.001), role functioning (*p* < 0.05), social functioning (*p* < 0.001), pain (*p* < 0.001), and increased diabetes distress levels (*p* < 0.001). Education was not related to HRQOL only slight difference was observed among low education status women with diabetes distress levels. Multivariate analysis also showed association as age with health perception and mental health, gender with social and physical functionings, and marital status with pain, self-care behavior, and distress levels. Findings suggested that physical functioning has strong predictor effect on health perception, mental health, pain, distress levels, and social functioning. Tables 2 and 3 present the results of multivariate models.

### 4.5. Impact of DLC and Diet on HRQOL

Women showed better mental health status with net benefits of the regimen than men (*p* < 0.001). Results showed that strong internal DLC is significantly related with less pain, improved social functioning, and reduced distress levels. While low significant association was found with beliefs in other DLCs on poor health perception (*p* < 0.05).

Regular diet showed significant association with poorer health perception and mental health. Women with proper diet management exhibits better perceived health, mental health, and reduce pain exposures.

### 4.6. Glycemic Control (HbA1c) and HRQOL

Earlier, we found that women with longer duration of diabetes and uncontrolled glycemic levels have impact on HRQOL factors; however, to evaluate the close relationship between glycemic levels and HRQOL, further analysis was made by factorizing the variable with three categories: consistent control (HbA1c < 6%), moderate control (HbA1c 6–8%), and poor control (HbA1c > 8%). Both pain (*p* < 0.01) and role functioning (*p* < 0.001) were found significant with the glycemic level. Findings showed no gender difference in the pattern suggesting that problems in role functioning and pain experiences were more frequent among poor glycemic control participants than the consistent control group. Thus, it strongly predicts that longer diabetes exposure is strongly related to poorer HRQOL. Tables 2 and 3 present the results of multivariate models.

### 4.7. Physical Functioning and HRQOL

The ratio between reported good to impaired physical functioning among participants was 1.8 : 1. The percentage variances were calculated to determine the contributions of each predictor variable in predicting health perception, mental health, and distress levels among normal and limited physical functioning (Table 4). In participants with good physical functioning (regardless of gender differences), the most predicting variable was diabetes-specific psychosocial factors both in health perception and mental health. However, women showed significantly higher association with health perception (15% versus 13% men, *p* < 0.001) and mental health (13% versus 11% men, *p* < 0.001) in diabetes-specific psychosocial factors. Thus, among women alone diabetes-related specific and psychosocial factors explained 15% and 13% of variations in HRQOL extents, respectively. However, participants with impaired physical functioning both health factors and diabetes-specific psychosocial factors among women with 15% and 14% in health perception. While social support was the only strongest predictor in mental health of women (13%). In contrast, among men participants with physical impairment, variance predicted with health factors was only 13% (*p* < 0.01) in health perception and 11% with social support in mental health.

### 4.8. Diabetes Distress Levels (PAID) and Physical Impairment

In multivariate analysis, PAID values were added to the model, it predicts regardless of physical functioning (impaired/good) and gender (man/woman) significantly and strongly predicts the effect of high distress levels with poorer HRQOL in health perception (*p* < 0.001), mental health (*p* < 0.017), social functioning (*p* < 0.011), and pain experiences (*p* < 0.012) (Table 4).

## 5. Discussion

The outcome measures of this study assessed HRQOL, which is more responsive to disease-specific psychosocial factors, social support, disease outcome, and disease distress levels than subjective factors. This study has reported a low nonresponse rate about 14.95% mainly with tediousness of the questionnaires and blood testing procedure, not influenced on the internal validity of the study. To be absolutely sure, we evaluated the demographic factors for nonresponse bias and it showed no significant findings. However, this does not apply to other factors like health-related and disease-specific psychosocial factors. Thus, demographic factors were controlled in the multivariate modelling procedure to increase the external validity of the study.

The study findings are consistent with the previous published literature, older participants [10–19, 23, 24] and respondents with low educational status [18–20, 23, 24] showed poorer HRQOL and social well-being. However, analysis showed that the health-related factors and disease-specific psychosocial factors mediated the association between sociodemographic and HRQOL; therefore, after adjusting these factors, there was no significant association reported between demographic and HRQOL. Several studies reported the role of cognitive factors in mediating the effect between demographic factors with health outcomes [25]. None of the previous literature reported the gender differences in multivariate analysis; only the present study focused on the effect of multidimensional factors on HRQOL including distress levels and also determined the gender differences in the five-relative domains.

Physical and social functionings are the determinants for the good perceived health status as well as well-being of the patient [17, 25]. The present study supports the literature and reports that both physical and social functionings had strong influence on perceived health, mental health, role functioning, pain, and also diabetes distress levels. Impact was also found with glycemic levels and duration of diabetes exposure but to lesser extent. Gender differences revealed significant findings with women, physical functioning, and diabetes distress levels compared to men. Further analysis also showed that reported diabetes comorbidities significantly lower the social and health well-being among patients with IDDM; these findings support the earlier findings as well [15–18, 22]. Glycemic control did not relate to HRQOL (all five dimensions) in the regression model; however, slight association was found among women in health perception and distress levels than men. Also, in poorer to consistent glycemic control groups, more frequent problems were reported with role functioning, physical functioning, pain, and high distress levels.

Social support, either general social support or diabetes-specific social support, reported beneficial outcomes among diabetes patients for lowering depression levels [19, 26]. Similarly, self-care behavior/self-efficacy also showed improvement in patient health-related outcomes and perceived diabetes distress status [27]. In concordance to these literature findings, our study reported better perceived HRQOL (except mental health and pain) with perceived adequacy of general social support. Lower distress levels also associated with perceived adequacy of support. In addition to these, we further analyzed the diabetes-specific social support factors and found significant association in modifying HRQOL among participants of this study. Improved social functioning, better health perception and mental health, and low distress levels were highly associated with strong perceived self-care behavior participants. Findings also showed that participants reported to receive strong self-efficacy/care related support were better well-bring in health perception, mental health, and low distress levels, even in controlling the general support factors. Both SMBG and diet showed marginal impact on HRQOL; however, gender differences revealed strong association of diet with women and SMBG among men participants. Usually diabetic diet (DASH) considered to be more public self-care than glucose self-monitoring, as diet significantly related to many more psychosocial factors. Sometime diabetic-specific diet may cause unwanted experiences, [25, 27] and this can heighten the role of illness in participant's life.

The health-related outcomes were generally better reported with perceived disease control [18, 19]. Also, patient education and counselling improves both health literacy and self-care behavior that will be beneficial to disease management [26, 27]. The present study reported moderate association of diabetes-control beliefs to HRQOL, particularly related with pain experiences and diabetes distress levels. The findings of this study identified several patterns that are significantly modifying HRQOL dimensions among IDDM patients. Also debated the gender differences to individualize the care plan on patient-specific characteristics. With the presence of comorbidities, physical impairment generally presented with lower/poorer HRQOL and high distress levels. In contrast, glycemic/metabolic control levels are not modifying HRQOL or distress levels except women who only call for attention. Diabetes-specific psychosocial factors, self-efficacy behavior, and social feedback would be modified and improved with education and intervening counseling.

### 5.1. Limitations of the Study

1. Cross-sectional study design weakened the internal validity so the implication would be tentative rather than definitive.
2. Lacking medical criteria for secondary clinical parameters determining diabetes mellitus related to comorbid conditions; for example, renal profiling, liver functioning, BMI, and hematological findings. Age criterion further limits the proportion of patients' sample and reduces the study population to 5–10%.
3. SF-20 questionnaire measures pain and social functioning with a single domain, it would be better to use a more refined and detailed measurement tool for these dimensions.
4. Self-care reporting particularly with self-perception may prod the diabetics to answer normatively.

## 6. Conclusion

This study concluded that there are various factors that affect the HRQOL among insulin-dependent diabetes patients. Women exhibit extensive and significant patterns with health-related factors and diabetes-specific psychosocial factors (self-efficacy, social support, and DLC) to improve HRQOL. Health-specific factors were more important among participants with physical impairment (regardless of age and gender). Also, women have significantly high reported distress levels and low social functioning compared to men.

### 6.1. Practice Implications

Practical implications of this study are focused on the subjective terms of HRQOL, social support, and disease distress levels rather than medical terms only. Strength of this study is the construct and correlational patterns of QOL as a multidimensional concept. The study provides sufficient information to develop a care plan on the basis of personal resources and life circumstances for individualized therapy. Lastly, the data from this study develop, promote, and support self-efficacy to improve disease factors with patient's self-care behavior.

## Figures and Tables

**Table 1:** Participants' characteristics of type I diabetes (*n* = 853).

Characteristics	Men (*n* = 436)^—^ 51.1%	Women (*n* = 417)^—^ 48.9%	Difference *p* value
Age^∗^, mean (years) ± SD	34.7 ± 7.4	36.1 ± 6.4	0.001
Marital status, *n* (%)			
Single	41 (9.40)	24 (5.8)	0.643
Married	299 (68.6)	297 (71.2)	
Widow	71 (16.3)	29 (7.0)	
Separated	25 (5.7)	67 (16.1)	
Education status, *n* (%)			
Primary	80 (18.3)	22 (5.3)	0.021
Intermediate	129 (29.6)	69 (16.5)	
Secondary	201 (16.1)	207 (49.6)	
University	26 (6.0)	159 (38.1)	
Occupation, *n* (%)			
Government	95 (21.8)	101 (24.3)	0.77
Private	87 (20.0)	84 (20.1)	
Business	254 (85.2)	232 (55.6)	
Diabetes exposure, mean (years) ± SD	15.5 ± 4.3	12.9 ± 6.8	0.001
Glycosylated hemoglobin, mean ± SD	9.3 ± 0.8	8.9 ± 1.3	0.485
Comorbidity, *n* (%)			
Congestive heart failure	147 (33.7)	284 (68.1)	0.001
Hyperlipidemia	239 (54.8)	113 (27.1)	
Mild renal disease	11 (2.6)	7 (1.7)	
None	39 (8.9)	13 (3.1)	
Mental health^ł^, mean ± SD	5.73 ± 2.14	6.98 ± 1.71	0.013
Health perception^≠^, mean ± SD	4.11 ± 1.22	6.46 ± 1.53	0.001
Role functioning, *n* (%)			
Impaired	140 (32.1)	205 (49.2)	0.189
Normal	296 (67.9)	212 (50.8)	
Social functioning, *n* (%)			
Impaired	96 (22.0)	101 (24.2)	0.014
Normal	340 (78.0)	316 (75.8)	
Physical functioning			
Impairments	138 (31.7)	169 (40.5)	0.017
No impairments	298 (68.3)	248 (59.5)	
Pain, *n* (%)—yes	188 (43.1)	271 (64.9)	0.001
Problem areas in diabetes (PAID) (diabetes distress scale), mean ± SD^┼^	36.4 ± 11.83	48.1 ± 10.51	0.011

^∗^Age range = 20–63 years, ^ł^Range = 0–10, ^≠^Range = 0–10, ^┼^Range = (0–100), ^—^Participants scoring ≥ 40 predicts “emotional burnout” in contrast participants with drop to ≤10 indicative for denial.

**Table 2:** Hierarchical regression analysis to evaluate patterns of health perception, mental health, and PAID on HRQOL.

Characteristics	Health perception^1^ (*β*^2^)		Mental health^1^ (*β*^2^)		PAID^3^ (*β*^2^)	
	Men	Women	Men	Women	Men	Women
Age	−0.15^∗^	−0.13^∗^	−0.05	−0.03	0.05	0.09^∗^
Marital status	−0.08	−0.05	0.03	0.08^∗^	0.02	0.07
Education	0.07Δ*R*^2^ = 0.13	0.09 Δ*R*^2^ = 0.11	0.09 Δ*R*^2^ = 0.10	0.09 Δ*R*^2^ = 0.010	0.10 Δ*R*^2^ = 0.09	0.15 Δ*R*^2^ = 0.12^∗∗^
Occupation	0.031	0.054	−0.02	−0.09	0.12^∗∗^	0.06
Diabetes exposure	−0.02	0.18^∗∗^	−0.12^∗^	−0.19^∗∗∗^	−0.09	−0.08
Glycemic control (HbA1c)	−0.04^∗^	−0.011^∗^	0.07	0.11^∗^	−0.21^∗∗∗^	−0.35^∗∗∗^
Comorbidities	−0.21^∗∗∗^	−0.26^∗∗∗^	−0.13^∗∗^	−0.21^∗∗∗^	−0.02	0.09
Physical functioning	0.38 Δ*R*^2^ = 0.24^∗∗^	0.41 Δ*R*^2^ = 0.35^∗∗^	−0.12 Δ*R*^2^ = 0.11^∗∗^	−0.25 Δ*R*^2^ = 0.23^∗∗^	0.24 Δ*R*^2^ = 0.19^∗∗^	0.29 Δ*R*^2^ = 0.25^∗∗∗^
Self-care behaviors						
Diet management	−0.09 Δ*R*^2^ = 0.01^∗^	−0.13 Δ*R*^2^ = 0.09^∗^	−0.03 Δ*R*^2^ = 0.00	−0.11 Δ*R*^2^ = 0.09^∗∗^	—	—
Self-monitoring blood glucose	0.13^∗^	0.17^∗∗^	0.02	−0.05	—	—
Social support						
Availability	−0.03	−0.06	−0.03	−0.17^∗∗^	−0.11^∗^	−0.19^∗∗^
Adequacy	0.14 Δ*R*^2^ = 0.10^∗^	0.19 Δ*R*^2^ = 0.17^∗∗^	−0.28 Δ*R*^2^ = 0.25^∗∗^	−0.31 Δ*R*^2^ = 0.29^∗∗^	0.21 Δ*R*^2^ = 0.20^∗∗^	0.29 Δ*R*^2^ = 0.26^∗∗∗^
Diabetes-related psychosocial factors						
Self-efficacy	0.24^∗∗^	0.39^∗∗∗^	0.16	0.24^∗∗∗^	−0.19^∗^	−0.31^∗∗∗^
Diabetes social support	0.14^∗^	0.17^∗∗∗^	0.11^∗^	0.35^∗∗∗^	−0.12	−0.23^∗∗^
DLC (others)	−0.09	−0.13^∗^	—	—	—	—
Severity	−0.03 Δ*R*^2^ = 0.02	−0.09 Δ*R*^2^ = 0.10^∗^	—	—	—	—
*R* ^2^/*R*^2^ adjusted	0.56/0.54^∗∗^	0.68/0.67^∗∗∗^	0.41/0.39^∗∗∗^	0.57/0.55^∗∗∗^	0.44/0.41^∗∗^	0.61/0.63^∗∗∗^

^1^Higher score indicates good health-related quality of life, ^2^Standardized coefficient, —Not entered in the stepwise procedure, ^3^Higher score indicates emotion distress. ^∗^*p* < 0.05, ^∗∗^*p* < 0.01, ^∗∗∗^*p* < 0.001.

**Table 3:** Hierarchical logistic regression analysis for social and role functioning and pain experiences among participants.

Characteristics	Pain		Impaired social functioning		Impaired role functioning	
	Odds (95% CI)		Odds (95% CI)		Odds (95% CI)	
	Men	Women	Men	Women	Men	Women
Age	1.03 (0.96–1.09)	1.05 (0.97–1.07)	1.08 (1.01–1.15)^∗^	1.11 (0.85–1.43)	1.05 (0.98–1.17)	1.07 (0.63–1.21)
Marital status	1.13 (1.01–1.19)	2.41 (1.23–4.70)^∗∗∗^	0.72 (0.28–1.86)	0.75 (0.41–1.00)	0.41 (0.21–0.65)	0.44 (0.13–0.73)
Education	1.01 (.57–1.61)	1.03 (0.67–1.55)	0.94 (0.47–1.86)	0.91 (0.56–1.28)	1.01 (0.71–1.32)	1.06 (0.81–1.19)
Occupation	0.79 (0.44–1.41)	0.83 (0.59–1.47)	0.30 (0.12–0.63)	0.47 (0.17—0.98)	1.36 (1.10–1.29)	1.39 (1.01–1.93)^∗^
Diabetes exposure	1.00 (0.94–1.06)	1.34 (1.13–2.61)^∗∗^	0.95 (0.79–1.01)	0.98 (0.62–1.32)	0.99 (0.73–1.29)	0.88 (0.39–0.99)
Glycemic control (HbA1c)	0.98 (0.82–1.17)	1.27 (1.11–1.98)^∗^	1.13 (0.86–1.45)	1.31 (1.11–1.99)^∗^	0.98 (0.67–1.36)	0.81 (0.24–1.11)
Comorbidities	1.01 (0.91–0.19)	1.43 (1.13–2.86)^∗∗^	2.32 (1.37–3.89)^∗^	3.76 (2.01–5.77)^∗∗^	1.41 (0.93–1.83)	1.67 (1.10–2.21)^∗∗^
Physical functioning	6.14 (4.11–13.98)^∗∗^	7.86 (4.23–14.49)^∗∗^	34.51 (10.73–49.46)^∗^	39.64 (10.11–43.4)^∗∗^	7.07 (2.81–14.37)^∗∗^	8.01 (2.96–18.69)^∗∗∗^
Self-care behaviors						
Diet management	2.33 (1.26–5.44)^∗^	6.23 (4.14–13.81)^∗∗^	—	—	—	—
Self-monitoring blood glucose	7.27 (5.15–15.48)^∗∗^	2.01 (0.86–3.16)	—	—	—	—
Social support						
Availability	0.96 (0.84–1.24)	1.01 (0.85–1.07)	1.07 (0.96–1.18)	1.13 (0.85–1.43)	1.09 (0.79–1.73)	1.18 (1.01–1.44)^∗^
Adequacy	0.89 (0.76–1.33)	0.88 (0.71–1.49)	0.95 (0.82–1.67)	0.97 (0.81–1.23)	0.89 (0.43–1.21)	0.93 (0.66–1.00)
Diabetes-related psychosocial factors						
Self-efficacy	—	—	0.79 (0.48–0.99)	0.81 (0.51–1.03)	—	—
Diabetes social support	0.91 (0.43–1.19)	0.80 (0.54–1.13)	—	—	0.77 (0.61–0.99)	0.84 (0.55–1.01)
DLC (others)	—	—	—	—	—	—
Problems areas in diabetes (PAID)						
≥40 points (distress burnout)	1.43 (1.13–1.89)^∗∗^	1.27 (1.01–1.94)^∗∗^	4.36 (2.19–8.36)^∗∗∗^	5.29 (3.38–10.42)^∗∗∗^	10.11 (4.62–21.11)^∗∗∗^	15.38 (5.32–23.3)^∗∗∗^

^—^Not entered in the stepwise procedure. ^∗^*p* < 0.05; ^∗∗^*p* < 0.01; ^∗∗∗^*p* < 0.001. N.B.: age: 40<, >40; marital: currently married/not; education: secondary<, >secondary; occupation: government, nongovernmental; diabetes exposure: <10, >10 years; glycemic control: <7, >7; comorbidities: yes/no.

**Table 4:** Variance (%) explained for health perception and mental health predictor^1^ groups on reported physical functioning.

Characteristics	No physical impairment (*n* = 546) %		Physical impairment (*n* = 307) %	
	Men (*n* = 298)	Women (*n* = 248)	Men (*n* = 138)	Women (*n* = 169)
*Health perception*				
Demographics	3^∗^	4^∗∗^	3	4^∗^
Health factors	4^∗^	3^∗^	13^∗∗^	15^∗∗∗^
Self-care behaviors	7^∗∗∗^	6^∗∗^	6^∗^	8^∗∗^
Social support	3	8^∗∗∗^	7	4
Diabetes-specific psychosocial factors	13^∗∗∗^	15^∗∗∗^	1	14^∗∗∗^
*Mental health*				
Demographics	1	3^∗^	4	4
Health factors	3^∗^	4^∗∗^	3	4
Social support	8^∗∗^	9^∗∗∗^	11^∗∗∗^	13^∗∗^
Diabetes-specific psychosocial factors	11^∗∗∗^	13^∗∗∗^	6^∗^	5^∗^
Self-care behaviors	1	0	8^∗^	3
*Diabetes distress (PAID)*	10^∗∗^	13^∗^	11^∗∗∗^	15^∗∗∗^

^1^Difference between *R*^2^ of the complete model: *R*^2^ of the model (after removing the predictor group). Demographics: age, marital status, education, and occupation. Health factors: diabetes exposure, glycemic control (HbA1c), comorbidities, and physical functioning. ^∗^*p* < 0.05, ^∗∗^*p* < 0.01, ^∗∗∗^*p* < 0.001.

## Data Availability

The data used to support the findings of this study are available from the corresponding author upon request.
